# Relationship of computed tomography perfusion and positron emission tomography to tumour progression in malignant glioma

**DOI:** 10.1002/jmrs.37

**Published:** 2014-01-16

**Authors:** Timothy P C Yeung, Slav Yartsev, Ting-Yim Lee, Eugene Wong, Wenqing He, Barbara Fisher, Lauren L VanderSpek, David Macdonald, Glenn Bauman

**Affiliations:** 1 London Regional Cancer Program, London Health Sciences CentreOntario, Canada, N6A 4L6; 2 Robarts Research Institute, The University of Western OntarioOntario, Canada, N6A 5B7; 3 Department of Medical Biophysics, The University of Western OntarioOntario, Canada, N6A 5C1; 4 Department of Oncology, The University of Western Ontario, London Health Sciences Centre, London Regional Cancer ProgramOntario, Canada, N6A 4L6; 5 Department of Medical Imaging, The University of Western Ontario, London Health Sciences Centre, Victoria HospitalOntario, Canada, N6A 5W9; 6 Lawson Health Research Institute, St. Joseph's Health Care LondonOntario, Canada, N6A 4V2; 7 Department of Physics and Astronomy, The University of Western OntarioOntario, Canada, N6A 3K7; 8 Department of Statistical and Actuarial Sciences, The University of Western OntarioOntario, Canada, N6A 5B7; 9 Department of Clinical Neurological Sciences, The University of Western Ontario, London Health Sciences Centre, University HospitalOntario, Canada, N6A 5A5

**Keywords:** 18-Fluorodeoxyglucose PET, contrast enhancement, CT perfusion, malignant glioma, tumour progression

## Abstract

**Introduction**This study aimed to explore the potential for computed tomography (CT) perfusion and 18-Fluorodeoxyglucose positron emission tomography (FDG-PET) in predicting sites of future progressive tumour on a voxel-by-voxel basis after radiotherapy and chemotherapy.

**Methods**Ten patients underwent pre-radiotherapy magnetic resonance (MR), FDG-PET and CT perfusion near the end of radiotherapy and repeated post-radiotherapy follow-up MR scans. The relationships between these images and tumour progression were assessed using logistic regression. Cross-validation with receiver operating characteristic (ROC) analysis was used to assess the value of these images in predicting sites of tumour progression.

**Results**Pre-radiotherapy MR-defined gross tumour; near-end-of-radiotherapy CT-defined enhancing lesion; CT perfusion blood flow (BF), blood volume (BV) and permeability-surface area (PS) product; FDG-PET standard uptake value (SUV); and SUV:BF showed significant associations with tumour progression on follow-up MR imaging (*P* < 0.0001). The mean sensitivity (±standard deviation), specificity and area under the ROC curve (AUC) of PS were 0.64 ± 0.15, 0.74 ± 0.07 and 0.72 ± 0.12 respectively. This mean AUC was higher than that of the pre-radiotherapy MR-defined gross tumour and near-end-of-radiotherapy CT-defined enhancing lesion (both AUCs = 0.6 ± 0.1, *P* ≤ 0.03). The multivariate model using BF, BV, PS and SUV had a mean AUC of 0.8 ± 0.1, but this was not significantly higher than the PS only model.

**Conclusion**PS is the single best predictor of tumour progression when compared to other parameters, but voxel-based prediction based on logistic regression had modest sensitivity and specificity.

## Introduction

In malignant glioma, over 80% of tumour progression occurs within 2 cm of the original tumour site after radiotherapy and concurrent and adjuvant temozolomide chemotherapy.^1^ Radiological assessment of tumour progression is primarily based on gadolinium-enhanced magnetic resonance (MR) or iodinated contrast-enhanced computed tomography (CT).^2^ An increase in the size of the contrast-enhancing lesion is one of the criteria for diagnosing progression.^2^ However, early changes in contrast enhancement after treatment lack utility in identifying active tumour sites that are likely to persist after treatment.

Functional imaging is gaining popularity as it has demonstrated utility in differentiating tumour grade, predicting treatment response and survival and in identifying active tumour after treatment prior to progression.^3^ Pre-treatment functional imaging measurements of tumour perfusion and metabolism have been shown to predict survival in newly diagnosed and recurrent malignant glioma.^4^–^7^ Similarly, changes in blood flow (BF) and blood volume (BV) during radiotherapy have been shown to be predictive of survival.^8^,^9^ The use of multiparametric imaging data may have an added value for predicting survival. For example, tumour metabolism and BF were combined into a metric called the metabolism-flow ratio, and this was found to be predictive of poor survival in pancreatic cancer.^10^ Metabolism and BF are usually tightly coupled in normal tissue; however, an increase in metabolism in the presence of low BF may be indicative of a tumour's adaptation to hypoxia.^11^ This suggests that the metabolism-flow ratio may be a valuable biomarker of hypoxia, which is associated with treatment resistance.

CT perfusion can simultaneously measure BF, BV and permeability-surface area product (PS) in brain tumours,^12^ and 18-Fluorodeoxyglucose positron emission tomography (FDG-PET) is in routine clinical use for measuring tumour metabolism. The combination of FDG-PET and CT perfusion using a hybrid PET/CT scanner is a practical technique for assessing metabolism and perfusion in quick succession. This can also be used to calculate the metabolism-flow ratio, which circumvents the need for specialised synthesis capabilities to produce hypoxia tracers such as 18-Fluoro-azomycin arabinoside.

No functional imaging studies have been reported near the completion of radiotherapy as an attempt to detect residual malignant gliomas. This study investigated the associations between the pre-radiotherapy gross tumour as defined on contrast-enhanced MR, near-end-of-radiotherapy enhancing lesion as defined on contrast-enhanced CT, near-end-of-radiotherapy CT perfusion and FDG-PET parameters with post-treatment MR-defined tumour progression. We then assessed the value of using these imaging parameters for predicting post-treatment MR-defined tumour progression. Therefore, the goal of this study was to assess whether functional imaging has the potential in predicting sites of future progressive tumour on a voxel-by-voxel basis after radiotherapy and chemotherapy. This can help identify biologically significant volumes of disease present at the end of conventional radiotherapy that could serve as a target for treatment intensification.

## Methods

### Patients

All study procedures were approved by Institutional Research Ethics Board. Patients with newly diagnosed malignant glioma were prospectively recruited to the study with informed consent prior to the last week of radiotherapy. The inclusion criteria were: (1) at least 18 years of age, (2) histologically confirmed malignant glioma, (3) Karnofsky Performance Status ≥60, (4) no previous cranial radiotherapy and (5) pre-radiotherapy MR were performed within 12 weeks of radiotherapy treatment planning. Recurrent gliomas, multiple intracranial lesions, or patients that were not suitable for radical radiotherapy (40–60 Gy in 15–30 fractions) were excluded.

### Multi-modality imaging schedule

After surgery but before radiotherapy, each patient underwent a standard MR imaging comprised of at least one series of T1- and T2-weighted images using a 1.5 T MR scanner (Signa Excite, General Electric Medical Systems, Milwaukee, WI). This MR scan was performed within 12 weeks of the treatment planning CT. Contrast-enhanced T1-weighted images were acquired using a fast spoiled gradient echo sequence (repetition time = 10 msec, echo time = 4 msec and flip angle = 15°) after an intravenous injection of gadopentetate dimeglumine (0.2 mL/kg; Magnevist; Berlex Laboratories, Wayne, NJ).

A FDG-PET and CT perfusion scan was performed during the final week of radiotherapy using a hybrid PET/CT scanner (Discovery VCT, General Electric Healthcare, Waukesha, WI). After an intravenous injection of FDG (385 MBq), positron emission data were collected in list mode for 60 min and binned into 5 min frames in the last 40 min. Each PET data set consisted of 47 axial PET images with a 3.26 mm slice thickness and a 26 cm axial field of view (FOV).

The FDG-PET CT attenuation correction maps in the FDG-PET study were used to select eight 5 mm sections to maximally cover the tumour for a 150 sec two-phase CT perfusion scan. A non-ionic contrast bolus (Omnipaque, General Electric Healthcare, Princeton, NJ, 300 mg iodine per mL, 0.8 mL/kg of body weight) was injected at a rate of 2–4 mL/sec at 3–5 sec before the start of the scan. While the selected brain sections were scanned continuously for 45 sec and the images reconstructed at 1-sec intervals during the first phase, the same sections were scanned once every 15 sec for seven times during the second phase. The scanning parameters for both phases were 80 kVp, 190 mA, 1 sec per rotation and a 25-cm FOV.

Post-radiotherapy follow-ups were both clinical and radiological. Routine MR images were obtained at 4–6 weeks post-radiotherapy and every 3 months thereafter, and were reviewed by a neuro-oncologist or radiation oncologist. Tumour progression was defined as a continual enlargement of new enhancing lesion (without subsequent resolution) on serial MR imaging along with deterioration of clinical signs and symptoms.^2^

### Image processing

CT perfusion images were analysed using the prototype version of CT Perfusion 4D (Advantage Windows, GE Healthcare, Waukesha, WI). Arterial and venous time-attenuation curves were measured from an anterior cerebral artery and the posterior superior sagittal sinus respectively. BF, BV and PS maps were calculated by deconvolving the arterial time-attenuation curve with the tissue time-attenuation curve from 2 × 2 voxel blocks of CT images.^12^ The CT images were averaged together to produce average CT images, which have better grey and white matter contrast than conventional contrast-enhanced CT images.

Software provided with the PET/CT scanner was used to correct the PET emission data for random and scatter coincidences, dead time and attenuation. PET images from the last 5 min bin were rigidly co-registered with the average CT using the 3D Slicer software.^13^ The registered PET voxel values were converted to standard uptake values (SUV) corrected for body surface area.^14^ SUV maps were divided by the BF maps to generate SUV:BF ratio maps.

Gadolinium-enhanced T1-weighted MR images from pre-radiotherapy and at progression were rigidly co-registered with the near-end-of-radiotherapy average CT. All images (MR and average CT) and functional maps (CT perfusion and PET SUV) were then resampled to 170 × 170 voxels to minimise registration error. From here on, the contrast-enhancing lesion in the pre-radiotherapy T1-weighted MR lesion is the gross tumour, the contrast-enhancing lesion on the average CT acquired at the last week of radiotherapy is the near-end-of-radiotherapy enhancing lesion, and the contrast-enhancing lesion on follow-up T1-weighted MR at the time of progression is the progressive tumour. Contours around the pre-radiotherapy gross tumour, near-end-of-radiotherapy enhancing lesion and progressive tumour were delineated by a radiation oncologist (G. B.). Table 1 summarises the labelling and time of acquisition of the images.

**Table 1:** Summary of images acquired in this study.

Name	Type of image(s)	Image signal	Time of acquisition
Gross tumour	T1-weighted MR	Gadolinium enhancement	Post-surgery/biopsy and pre-radiotherapy (must be within 12 weeks of treatment planning CT)
Near-end-of-radiotherapy enhancing lesion	Average CT	Iodine enhancement (HU)	Last week of radiotherapy
BF	BF map	mL/min per 100 g	Last week of radiotherapy
BV	BV map	mL/100 g	Last week of radiotherapy
PS	PS map	mL/min per 100 g	Last week of radiotherapy
PET	SUV map	FDG uptake	Last week of radiotherapy
Progressive tumour	T1-weighted MR	Gadolinium enhancement	Time of progression based on routine clinical and radiological follow-up assessments (4–6 weeks post-radiotherapy and every 3 months after)

MR, magnetic resonance; CT, computed tomography; BF, blood flow; BV, blood volume; PS, permeability-surface area product; PET, positron emission tomography; SUV, standard uptake value; FDG, 18-Fluorodeoxyglucose.

### Logistic regression

A custom MATLAB (MathWorks Incorporated, Natick, MA) program was developed for this analysis. A 2-cm bounding box around the union of the pre-radiotherapy gross tumour and the progressive tumour was created to reduce the amount of information for analysis for each patient. Major blood vessels, surgical cavities, ventricles and voxels outside the bounding box were excluded. Each voxel inside the bounding box was assigned three binary statuses: pre-radiotherapy gross tumour (yes/no), near-end-of-radiotherapy enhancing lesion (yes/no) and progressive tumour (yes/no). Logistic regression was used to assess the association between tumour progression and the following sets of independent imaging variables: (1) pre-radiotherapy gross tumour, (2) near-end-of-radiotherapy enhancing lesion, (3) BF, BV, PS and SUV and (4) BV, PS, SUV:BF.^15^ The last regression was used to investigate the association between tumour progression and SUV:BF as a marker of hypoxia. The strength of association between each parameter and progressive tumour status was evaluated by the odds ratio. Odds ratios were converted to probabilities of recurrence for easier interpretations.

### Cross-validation

Leave-one-out cross-validation was used to assess the value of using different imaging parameters to predict tumour progression.^16^ Each round of cross-validation used one patient data set as the validation set and the data sets from the other patients as the training set. Probability maps of progression for an individual patient (i.e. the validation set) were calculated using the logistic regression equation from the training set. We first considered seven single variable models: (1) Pre-radiotherapy gross tumour, (2) Near-end-of-radiotherapy enhancing lesion, (3) BF, (4) BV, (5) PS, (6) SUV and (7) SUV:BF. We then considered two multivariate models: (1) BV, PS, SUV:BF and (2) BF, BV, PS and SUV. Using receiver operating characteristic (ROC) analysis, the area under the ROC (AUC) curve was calculated. The optimal sensitivity and specificity was calculated using the Youden Index, which is the probability threshold that maximises the sum of sensitivity and specificity for all possible probabilities from 0% to 100%.^16^

### Statistical analysis

Pre-radiotherapy gross tumour, near-end-of-radiotherapy enhancing lesion and progressive tumour volumes within the 4 cm CT perfusion scan length were compared using the Wilcoxon signed-rank test. AUCs of the different logistic models were compared using the Friedman test followed by the Wilcoxon signed-rank test. Models with AUCs that are significantly higher than the AUCs of the pre-radiotherapy gross tumour and end-of radiotherapy enhancing lesion were reported. All statistical tests were done using SPSS version 19.0 (SPSS Incorporated, Chicago, IL), and *P*-values <0.05 were considered significant.

## Results

Ten patients with newly diagnosed malignant glioma were prospectively recruited to the study between the years 2008 and 2011. After surgery, all patients underwent radiotherapy (60 Gy in 30 fractions) with concurrent and adjuvant temozolomide chemotherapy. Patient characteristics are listed in Table 2. Tumour progression with no evidence of necrosis was histopathologically confirmed in two patients (Patient 9 and 10 in Table 2); the remaining patients had tumour progression defined on clinical and imaging grounds only.

**Table 2:** Patient characteristics.

Patient no.	Age	WHO grade	Tumour location	Type of resection	Months between radiotherapy and appearance of progressive tumours	Site of tumour progression	Treatment	Steroid use	Survival status
1	36	3	Left frontal temporal	Partial	8.6	1In-field	RT, TMZ	Yes	Deceased
2	47	4	Left parietal	Near-total	12.0	In-field	RT, TMZ	Yes	Deceased
3	55	4	Left temporal	Partial	2.3	In-field	RT, TMZ	Yes	Deceased
4	37	3	Left frontal temporal	Partial	8.4	In-field and 2Out-of-field	RT, TMZ	No	Deceased
5	50	4	Left frontal lobe	Near-total	5.0	In-field	RT, TMZ	Yes	Deceased
6	71	4	Right temporal parietal	Biopsy	2.1	In-field	RT, TMZ	Yes	Deceased
7	71	4	Left frontal	Biopsy	4.3	In-field	RT, TMZ	Yes	Deceased
8	61	4	Left posterior frontal parietal inter-axial tumour	Near-total	14.8	In-field	RT, TMZ	Yes	Deceased
9	60	4	Left frontal parietal	Partial	11.0	In-field	RT, TMZ	Yes	Alive
10	54	4	Left temporal	Biopsy	15.6	In-field	RT, TMZ	Yes	Alive

WHO, World Health Organisation; RT, radiotherapy; TMZ, concurrent + adjuvant temozolomide.

1 In-field = within 2 cm of the primary contrast-enhancing tumour.

2 Out-of-field = beyond 2 cm of the primary contrast-enhancing tumour.

**Figure 1:**
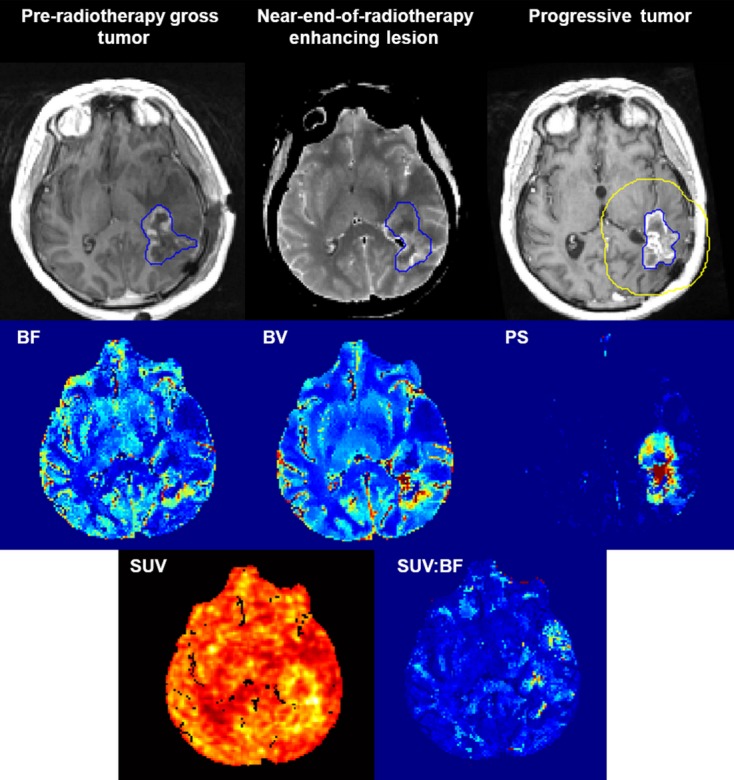
Pre-radiotherapy gross tumour (T1-weighted MR), near-end-of-radiotherapy enhancing lesion (averaged CT) and progressive tumour (T1-weighted MR); and the corresponding parametric maps of blood flow (BF), blood volume (BV), permeability-surface area (PS) product, standard uptake value (SUV) and SUV:BF acquired using CT perfusion and FDG-PET. Blue outlines show the contrast-enhancing lesions delineated by a radiation oncologist. Yellow outline is the 2-cm bounding box that was set for performing logistic regression. MR, magnetic resonance; CT, computed tomography; FDG-PET, 18-Fluorodeoxyglucose positron emission tomography.

The mean volumes ±standard deviation (SD) of pre-radiotherapy gross tumour, near-end-of-radiotherapy enhancing lesion and the progressive tumour within the CT perfusion scan volume were 10.8 ± 11.1, 14.1 ± 20.8 and 16.1 ± 18.0 cm^3^ respectively. The differences in volumes were not significant (*P *>* *0.05). Figure 1 displays the images of patient 3.

### Voxel-based logistic regression

When compared with normal brain tissue, the pre-radiotherapy gross tumour and the near-end-of-radiotherapy enhancing lesion had odds ratios of 12.7 (95% confidence interval [CI], 12.1–13.4) and 43.3 (95% CI, 41.4–45.3), respectively, for tumour recurrence. These odds can be expressed as recurrence probabilities of 54.4% and 69.8% respectively. Multivariate logistic regression showed that the odds of tumour progression increased with lower BF, BV and SUV; and with higher PS and SUV:BF (*P *<* *0.0001) (Table 3).

**Table 3:** Multivariate logistic regression.

					95% confidence interval		
Model	Parameter	*P*	1Regression coefficients (*β*)	Odds ratio (e^*β*^)	−95%	+95%	Probability (%)
1	BV	<0.0001	−0.15	0.86	0.84	0.87	7.1
	PS	<0.0001	0.41	1.51	1.49	1.52	11.9
	SUV:BF	<0.0001	2.63^2^	13.88	11.38	16.94	55.4
	Constant	<0.0001	−2.41	0.09	0.09	0.09	8.2
2	BF	<0.0001	−0.01	0.99	0.99	0.99	23.1
	BV	<0.0001	−0.07	0.93	0.91	0.95	22.1
	PS	<0.0001	0.39	1.48	1.47	1.50	31.0
	SUV	<0.0001	−0.93	0.39	0.37	0.41	10.7
	Constant	<0.0001	−1.19	0.30	0.29	0.32	23.3

BV, blood volume; PS, permeability-surface area product; SUV, standard uptake value; BF, blood flow.

1 Degrees of freedom = 128,330.

2 An increase in SUV:BF value by 0.01 was associated with an odds ratio = 1.03; this corresponded to a probability of 8.4%.

### Cross-validation

**Figure 2:**
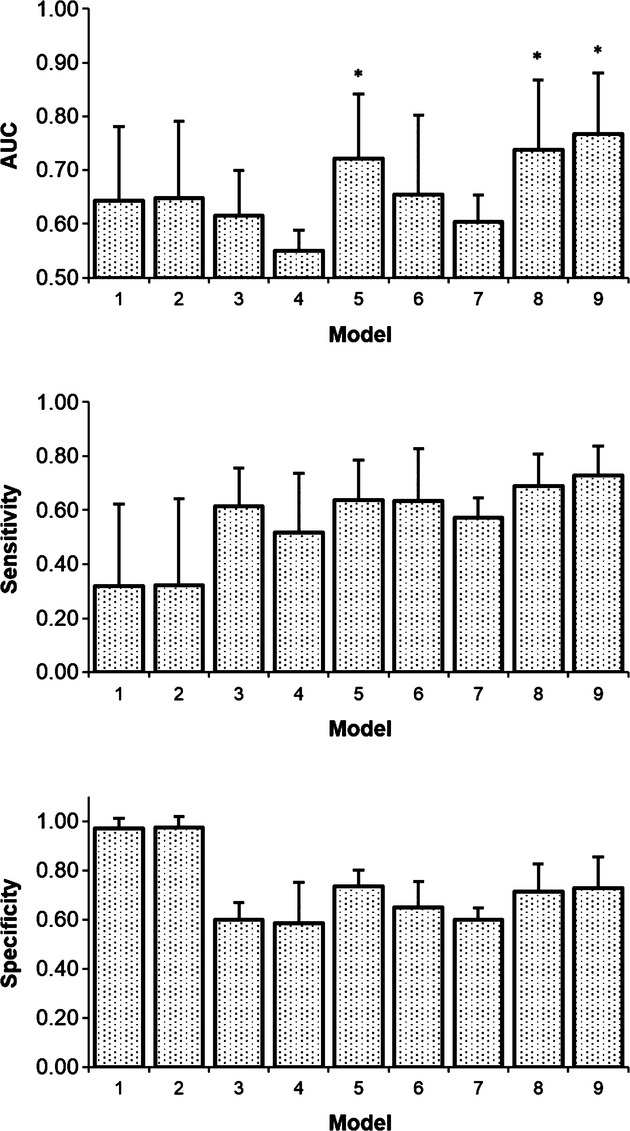
Area under the operating characteristic curve (AUC) (top), sensitivities (middle) and specificities (bottom) for the selected logistic regression models. Models with an AUC that is significantly higher than the pre-radiotherapy gross tumour and the end-of-radiotherapy enhancing lesion are indicated with an asterisk (*). 1, Pre-radiotherapy gross tumor; 2, End-of-radiotherapy enhancing lesion; 3, BF; 4, BV; 5, PS; 6, SUV; 7, SUV:BF; 8, BF, PS, SUV:BF; 9, BF, BV, PS, SUV.

**Figure 3:**
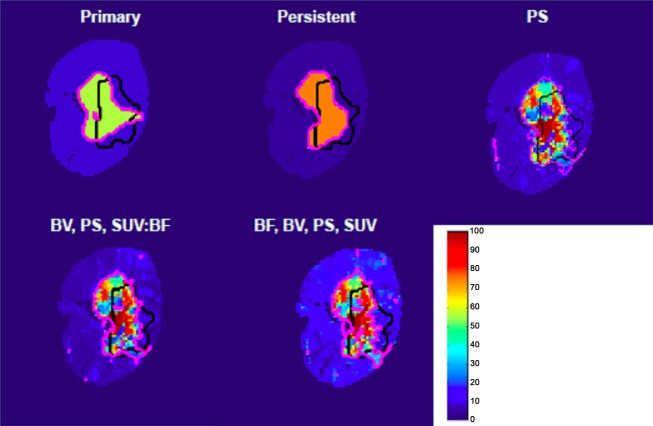
Probability of tumour progression (patient 3) within the 2-cm bounding box generated using the different logistic regression models. Black line outlines the boundary of the progressive tumour. Based on cross-validation, the magenta line delineates the region with the probability threshold that maximises the sum of sensitivity and specificity in predicting progression.

The AUCs for the three models (1) PS alone, (2) BV, PS, SUV:BF and (3) BF, BV, PS and SUV were 0.72 ± 0.12, 0.74 ± 0.13 and 0.77 ± 0.11 respectively. These AUCs were significantly higher than the AUCs of the pre-radiotherapy gross tumour and near-end-of-radiotherapy enhancing lesion (Friedman *P *<* *0.001; Wilcoxon signed-rank *P *≤* *0.03), which were 0.64 ± 0.14 and 0.65 ± 0.14 respectively. Although the AUCs of the two multivariate models were higher than that of PS, the differences were not statistically significant (*P *>* *0.05). Table 4 shows model pairs that are significantly higher than the pre-radiotherapy gross tumour and the end-of-radiotherapy enhancing lesion. The model using PS alone had a sensitivity and specificity of 0.6 ± 0.1 and 0.7 ± 0.1, respectively (Fig. 2). The model using BF, BV, PS and SUV had the highest combination of sensitivity and specificity (0.7 ± 0.1 and 0.7 ± 0.1). Probability maps of progression were generated using these logistic models, examples of probability maps for patient 3 are shown in Figure 3.

**Table 4:** Statistical differences in area under the receiver operating characteristics curve.

Model 1	Model 2	% Difference = (model 2−model 1)/(model 1)×100	±SD	*P*
Pre-radiotherapy gross tumour	PS	14.2	18.1	0.03
	BV, PS, SUV:BF	16.4	18.6	0.03
	BF, BV, PS, SUV	21.4	18.6	<0.01
End-of-radiotherapy enhancing lesion	PS	12.7	10.7	0.02
	BV, PS, SUV:BF	15.4	16.7	0.03
	BF, BV, PS, SUV	21.1	20.4	0.02
PS	BV, PS, SUV:BF	2.1	7.2	0.29
PS	BF, BV, PS, SUV	7.5	15.9	0.29

SD, standard deviation; PS, permeability-surface area product; BV, blood volume; SUV, standard uptake value; BF, blood flow.

## Discussion

We investigated whether near-end-of-radiotherapy CT perfusion and FDG-PET can add value to the assessment of malignant glioma in terms of predicting voxels that are likely to progress after treatment. PS alone was found to be the best predictor of tumour progression when compared to other CT perfusion and FDG-PET parameters; however, voxel-based prediction of progression had only modest sensitivity and specificity.

PS describes the unidirectional flow of contrast as it leaks from the intravascular space into the interstitial space (i.e. brain parenchyma).^12^ It is not the same as contrast enhancement because it quantitatively measures the rate of contrast extravasation. In this study, not only did PS show a significant association with progressive tumour but it had a higher AUC than the pre-radiotherapy gross tumour and near-end-of-radiotherapy enhancing lesion for identifying tumour voxels that are more likely to progress. This suggests that PS adds value to contrast enhancement in determining active tumour regions after treatment, and also provides corroborating evidence to support that PS is associated with tumour aggressiveness. PS can be a biomarker of tumour aggressiveness because of the underlying pathological aspect that it represents. PS has larger value in high-grade gliomas compared to low-grade gliomas,^17^ which is important for prognosis. It also showed correlations with aggressive phenotypes including microvascular cellular proliferation^18^ and the expression of pro-angiogenic genes.^19^ Finally, PS has been used to distinguish true tumour progression from treatment effect like treatment-induced necrosis.^20^

The observation that both lower BF and BV showed significant associations with tumour progression is noteworthy. A lower BF and BV may suggest resistance to therapy due to the presence of hypoxia. In support of this, dynamic susceptibility contrast MR studies at pre- and mid-radiotherapy showed that a drop in relative BF and BV were predictors of poor survival.^8^,^9^ However, the associations observed in this study were obtained from one time point only, and they do not suggest a causal relationship with progression.

We found that a lower SUV, when considering the entire tumour, is associated with an increased risk of progression. This is contrary to studies that showed an increased FDG uptake is associated with poor survival in patients at various stages of treatment^21^ or in patients with recurrent tumours.^7^,^22^ In these prior studies, FDG uptake in the hypermetabolic region was used to assess correlation with survival. This usually coincides with the contrast-enhancing region of the tumour where the perfusion is high; hence, an FDG delivery was unimpeded. Our results suggest that when considering the tumour as a whole, a lower FDG uptake is associated with higher odds of progression. A low FDG uptake does not necessarily infer a lack of metabolic demand. We instead showed a higher SUV:BF ratio was associated with an increased risk of recurrence, which suggests that a low SUV was due to poor delivery resulting in uncoupling between metabolism and perfusion. This is consistent with PET-based measure of SUV:BF in pancreatic cancer in which a higher SUV:BF was associated with poor survival.^10^ These observations provide evidence to support the Warburg Effect hypothesis.^10^,^11^ Cancer cells can maintain anaerobic respiration to keep up with its metabolic demand under hypoxic stress and can be therapeutically resistant. This could lead to tumour regions that remain active despite treatment.

Some limitations of this study must be considered. Although the AUCs of the multivariate models were higher than those of the univariate PS model, we were unable to demonstrate the statistical significance of multivariate models superiority. A larger sample size is required to determine whether (1) BF, BV, PS and SUV and (2) BV, PS and SUV:BF can provide more information than PS alone for identifying tumour regions that are likely to progress. Other radiotracers with a higher tumour-to-background ratio in the brain can improve the detection of residual tumour at the end of radiotherapy. For example, both 3′-Deoxy-3′-18F-fluorothymidine (FLT)^23^ and 11C-methionine PET^24^ were reported to be more sensitive than FDG in evaluating progressive tumours. Deformable registration could potentially improve the quality of registration. Most deformation of the tumour would occur from before and after surgery. This study used only post-surgery images; thus, physical deformation of the tumour is minimal. In addition, the majority of patients received dexamethasone during radiotherapy, which could decrease tumour permeability resulting in less oedema and hence less deformation.^25^ The use of dexamethasone can also decrease cerebral glucose metabolism.^26^,^27^ The effect of dexamethasone on glucose metabolism is global rather than localised; it is unlikely to affect the current results because the logistic regression is based on imaging data obtained from both the normal brain and tumour tissues. Finally, we focused our investigation on the tumour site and its immediate surroundings, the potential effects of radiation on normal brain tissue perfusion and metabolism were not investigated in detail. A small radiation dose-dependent relationship (<10%) was observed for both brain tissue metabolism and perfusion.^28^ We evaluated the relationship between normal brain tissue BF, BV and PS with radiation dose in seven patients and did not find a significant relationship (data not shown). Thus, the effect of radiation on normal tissue BF, BV and PS is unlikely to affect the current results.

## Conclusions

We developed a technique for voxel-wise analysis of CT perfusion and FDG-PET images acquired at the end of radiotherapy for patients treated for malignant glioma. On the basis of our analysis, near-end-of-radiotherapy PS was the best predictor of tumour progression on a voxel-by-voxel basis when compared to other CT perfusion and FDG-PET parameters. However, voxel-based analysis had only modest sensitivity and specificity. Exploration of these findings among a larger patient cohort would help confirm the value of PS imaging in predicting recurrence and in confirming the trend towards improved prediction with multivariate imaging data.

## Supporting Information

Video S1 A dynamic series of CT perfusion images from which the maps of blood flow (BF), blood volume (BV), and permeability-surface area (PS) product were calculated from.
